# Neuroinflammation and major depressive disorder: astrocytes at the crossroads

**DOI:** 10.3389/fncel.2024.1504555

**Published:** 2024-11-22

**Authors:** Melissa Puentes-Orozco, Sonia L. Albarracin, María Marcela Velásquez

**Affiliations:** ^1^Departamento de Nutrición y Bioquímica, Pontificia Universidad Javeriana, Bogotá, Colombia; ^2^Instituto de Genética Humana, Facultad de Medicina, Pontificia Universidad Javeriana, Bogotá, Colombia

**Keywords:** major depressive disorder, astrocytes, neuroinflammation, antidepressants, neuroplasticity

## Abstract

Major depressive disorder is a complex and multifactorial condition, increasingly linked to neuroinflammation and astrocytic dysfunction. Astrocytes, along with other glial cells, beyond their classic functions in maintaining brain homeostasis, play a crucial role in regulating neuroinflammation and neuroplasticity, key processes in the pathophysiology of depression. This mini-review explores the involvement of astrocytes in depression emphasizing their mediation in neuroinflammation processes, the impact of astrocytic dysfunction on neuroplasticity, and the effect of some antidepressants on astrocyte reactivity. Recent evidence suggests that targeting astrocyte-related signaling pathways, particularly the balance between different astrocytic phenotypes, could offer promising evidence for therapeutic strategies for affective disorders. Therefore, a deeper understanding of astrocyte biology may open the way to innovative treatments aimed at mitigating depressive symptoms by impacting both neuroinflammation and imbalances in neuroplasticity.

## 1 Introduction

Major depressive disorder (MDD) is a complex and debilitating mental health condition that affects millions of people worldwide. Increasing evidence suggests that its pathophysiology goes beyond the traditional monoaminergic hypothesis, pointing to the role of neuroinflammation and astrocytic dysfunction (Kouba et al., 2024). Astrocytes, a key type of glial cell in the central nervous system, play a very important role in maintaining brain homeostasis, regulating synaptic transmission, supporting metabolic functions, and controlling inflammatory responses (Gradisnik and Velnar, 2023). Neuroinflammation, often triggered by stress and immune activation, has been linked to disruptions in neuroplasticity, which are thought to contribute to the cognitive and emotional alterations associated with depressive states (Wang et al., 2017).

Astrocytes are important modulators of neuroplasticity since they intervene in synaptogenesis and neuronal survival. Dysfunctions in these cells have been associated with increased neuronal death in depressive disorders (Peng et al., 2015). Likewise, it has been emphasized that the balance between the neuroprotective or neurotoxic effects of astrocytes, in inflammatory brain environments, is crucial to determine resilience to depressive episodes. For this reason, reactive astrogliosis appears to exacerbate neuroinflammation and thus contribute to the chronicity of depression (Wang et al., 2017). Recent evidence suggests that interventions that aim to impact astrocytic regulatory pathways could restore neuroplasticity and reduce depressive symptoms, thus highlighting the potential of astrocytes as therapeutic targets (Liu et al., 2024).

Given the complex role of astrocytes in both neuroinflammation and neuroplasticity, understanding their contribution to the development and progression of MDD is crucial for the advancement of treatment strategies. This mini-review aims to explore the intricate involvement of astrocytes in depression by discussing three key areas: the role of astrocytes as mediators of neuroinflammation and its effects on depression, the relationship between astrocytic dysfunction and impaired neuroplasticity, and the impact of some antidepressant treatments on astrocyte function. Addressing these interconnected topics would be crucial to reveal the multifaceted role of astrocytes in the pathophysiology of depression, underscoring the need for further research to elucidate their involvement in neuroinflammatory processes their involvement in neuroinflammatory processes. A deeper understanding of astrocyte biology and its interplay with neuroinflammation could open new avenues for developing novel therapeutic strategies aimed at alleviating depressive disorders.

## 2 Major depressive disorder, neuroinflammation, and astrocytic function

Major depression disorder (MDD) is a multifactorial affective disorder and one of the leading causes of disease burden globally (Cosgrove et al., 2024). MDD has a significant psychosocial impact, with clinical manifestations that encompass cognitive, somatic, emotional, and psychomotor symptoms, which significantly compromise the independence and functionality of those affected (James et al., 2018; World Health Organization, 2021). As a complex disorder, its etiology involves the interaction of genetic and environmental factors, further modulated by epigenetic mechanisms (Li Z. et al., 2021). Several hypotheses have been raised about its pathogenesis, including imbalances in monoaminergic neurotransmission, deregulation of the hypothalamic–pituitary–adrenal (HPA) axis, reduction in neuroplasticity, and changes in functional connectivity. However, the complete pathophysiology of the disorder remains poorly understood, and none of these hypotheses alone sufficiently explain its multidimensional phenotype (Cui et al., 2024).

Recent evidence increasingly links inflammatory processes to stress-related disorders, including major depression (Milaneschi et al., 2021). Depression is often characterized by a persisting state of neuroinflammation, accompanied by elevated blood concentrations of pro-inflammatory cytokines, such as interleukin-1β (IL-1β), interleukin-6 (IL-6), tumor necrosis factor-*α* (TNF-α), and some acute phase proteins, like C-reactive protein (CRP) (Howren et al., 2009; Kim and Won, 2017; Maeng and Hong, 2019; Munshi et al., 2020). This inflammatory environment has been shown to impact brain regions involved in emotional and cognitive processing, including the hippocampus, amygdala, prefrontal cortex, and anterior cingulate cortex (ACC), increasing vulnerability to depression (Kim and Won, 2017). In this context, the role of glial cells, particularly astrocytes, has gained significant attention in depression research because of its involvement in the regulation of the inflammatory response within the central nervous system (Giovannoni and Quintana, 2020).

**Figure 1:**
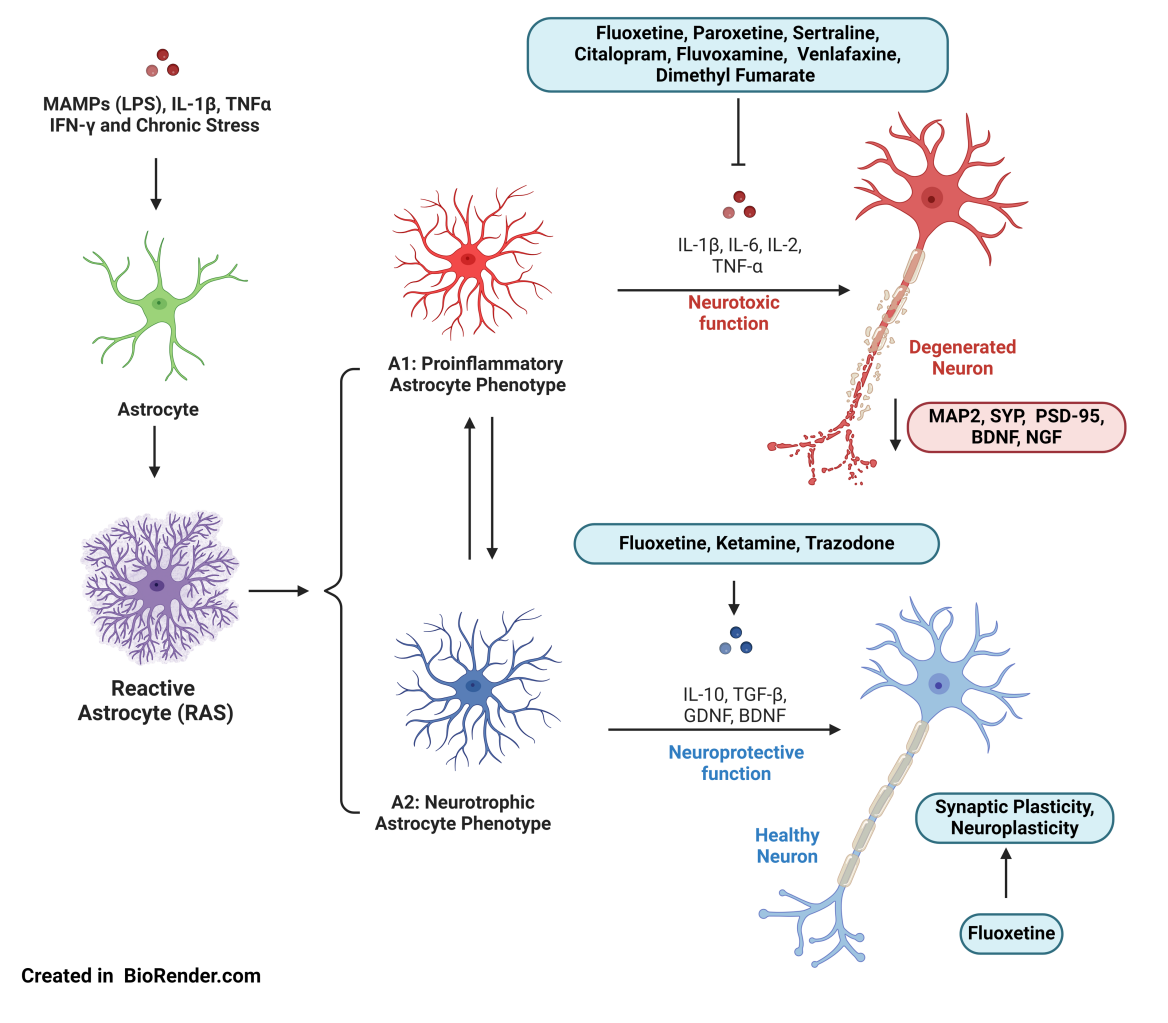
A1 and A2 astrocyte phenotypes in depression: the inflammatory environment, characterized by a chronic state of stress, increases cortisol levels and promotes reactive astrogliosis. At least two types of astrocytes are described: neurotoxic astrocytes (A1), which can induce neuronal death, and neurotrophic astrocytes (A2), which support neuronal survival and tissue repair. The pro-inflammatory A1 phenotype may contribute to synaptic plasticity alterations, reducing dendritogenesis and synaptic contact formation, which could explain behavioral changes in regions like the hippocampus. A2 astrocytes produce neurotrophic factors such as GDNF and BDNF, as well as anti-inflammatory molecules. Some antidepressants inhibit the production of pro-inflammatory cytokines and promote the release of neurotrophins, potentially regulating A1 astrocyte activity and encouraging the transition to the A2 phenotype. Figure was created using BioRender.com.

Beyond their traditional roles in metabolic cooperation with neurons, neurotransmitter uptake and catabolism (Bélanger et al., 2011; Yao et al., 2023), ammonium detoxification (Milaneschi et al., 2021), and antioxidant responses (Turati et al., 2020), astrocytes are also critical in regulating local cerebral blood flow (CBF) (Liu L. R. et al., 2020), homeostatic regulation, and maintaining the blood–brain barrier. Besides, studies in murine models have demonstrated that chronic stress induces astrocytic dysfunction, particularly in regions like the medial prefrontal cortex and hippocampus, correlating with behavioral changes typical of depressive phenotypes (Hao et al., 2020; Aten et al., 2023) (Figure 1). This dysfunction is linked to reduced expression of glucocorticoid receptors in astrocytes, which seem to be more sensitive to stress than neuronal receptors (Lu et al., 2022). Moreover, impaired astrocytic function can inhibit neurite outgrowth and destabilize glutamate release and reuptake, potentially leading to excitotoxicity (Planas-Fontánez et al., 2020; Zhang et al., 2024). One study found that the loss of astrocytes, rather than neurons, in the frontal cortex was sufficient to induce depressive-like behavior in rats, highlighting the critical role of astrocytes in the disorder’s pathophysiology (Banasr and Duman, 2008).

Reduced expression of the multiple endocrine neoplasia type 1 (Men1 or Menin) protein has been reported in astrocytes of animals exposed to lipopolysaccharide (LPS) or chronic unpredictable mild stress (CUMS). This was accompanied by increased activation of NF-κB and production of IL-1β, which led to depressive-like behaviors in these animals (Leng et al., 2018). Another study found that LPS exposure in mice promoted the transition of astrocytes into a neurotoxic (A1) state, also known as A1 reactive astrocytes (RAS), characterized by reduced brain-derived neurotrophic factor (BDNF) expression and elevated IL-1β and TNF-*α* levels (Wang et al., 2019).

Although the roles of different astrocyte activation states in MDD are not fully understood, reactive astrocytes persist in depression. Studies indicate that activation of the Nlpr3 inflammasome in microglia triggers neurotoxic astrocytes via the caspase-1 neuroinflammatory pathway in response to chronic stress. Notably, microglial Nlrp3 knockout has been shown to alleviate neuronal dysfunction caused by A1 astrocytes, both *in vitro* and *in vivo* (Li et al., 2022). Another study demonstrated that dehydrocorydaline could inhibit Nlpr3-mediated microglial activation and the release of TNF-α, IL-1α, and prostaglandin E2 (PGE2), thereby preventing A1 astrocyte activation and reducing depression-like behavior in C57BL/6 mice exposed to CUMS (Fang et al., 2022b). Furthermore, astrocyte pyroptosis has been observed during depression development in mouse models, with Nlpr3/Caspase-1/GSDMD-mediated pyroptosis contributing to both astrocyte loss in the hippocampus and behavioral changes (Li S. et al., 2021). Recent research also underscores the significance of calcium channels like Orai1 in astrocyte-driven inflammation and depression. The absence of Orai1 reduces inflammation-related Ca^2+^ signaling and diminishes inhibitory neurotransmission in the hippocampus (Novakovic et al., 2023). Another field of research supports a link between anti-inflammatory gut microbiota and depression. It seems that specific butyrate-producing bacteria are under-represented in patients compared to controls and that it could be associated with the typical chronic inflammation process of depression (Liu R. T. et al., 2020). However, it has not been described yet if there is any association between specific gut microbiota composition and astrocytic functioning under inflammatory conditions.

These findings suggest that neuroinflammation may play a critical role in both the onset and progression of depressive phenotypes (Yao et al., 2023). Thus, understanding the intricate functions of astrocytes in this context could prove essential for developing new therapeutic strategies for mood disorders.

## 3 Astrocyte phenotypes, neuroplasticity, and major depressive disorder

In response to changing conditions, astrocytes undergo a wide range of molecular, morphological, and functional adaptations, collectively known as reactive astrogliosis (Sofroniew, 2020) (Figure 1). Reactive astrocytes are highly heterogeneous and play a pivotal role in restoring homeostasis and repairing tissue damage (Hinkle et al., 2019). However, under certain circumstances, RAS can decrease adaptive neuronal plasticity (Sofroniew, 2020). Previous studies have identified two different types of RAS: neurotoxic astrocytes (A1), which can cause neuronal death, and neurotrophic astrocytes (A2), which promote neuronal survival and tissue repair (Clarke et al., 2018; Chang et al., 2023). The balance between A1 and A2 astrocytes influences synaptic function and neuroplasticity, determining neuronal outcomes under stress, and becoming a key component in the pathophysiology of depression (Hao et al., 2020).

Specifically, it has been suggested that the proinflammatory A1 phenotype may contribute to alterations in synaptic plasticity, dendritogenesis, spinogenesis, synaptogenesis, metabolism, brain volume, and behavioral functions. These changes could disrupt activity-dependent synaptic plasticity, which is crucial for proper neurotransmission and is known to be impaired in major depression. In several animal models of depression, two distinct patterns have emerged: increased plasticity in regions primarily involving amygdala neurons associated with the ventral emotional network and a decrease in neuronal plasticity in the dorsal executive system, which includes the hippocampus and large cortical areas (Kuhn et al., 2014). Consistent with these observations, several studies have shown reductions in the gray matter volume (GMV) in MDD, along with smaller hippocampal volumes, both of which are associated with worse depression scores (Cao et al., 2017; Kim et al., 2019). The largest global study on cortical structural alterations in MDD, performed under the ENIGMA (Enhancing Neuro Imaging Genetics through Meta-Analysis) consortium, identified widespread cortical abnormalities in patients with MDD compared to controls (Schmaal et al., 2017).

As previously suggested, activation of the microglial Nlpr3 inflammasome triggers the transition of astrocytes into the neurotoxic A1 state, impairing hippocampal synaptic plasticity. Li S. et al. (2021) measured the expression of neuronal nuclear antigen (NeuN), microtubule-associated protein 2 (MAP2), synaptophysin (SYP), and postsynaptic density-95 (PSD-95) in hippocampal neurons. They found that NeuN and MAP2, which indicate neuron number and dendritic morphology, were reduced, meanwhile, decreased levels of SYP and PSD-95 reflected impairments in synaptic plasticity and dendritic atrophy within the hippocampus (Li S. et al., 2021). Given the hippocampus’s critical connections with emotion-related areas such as the amygdala and anterior cingulate cortex, and its role in regulating the HPA axis via glucocorticoid receptors, it is particularly vulnerable to allostatic load, associated with chronic stress and elevated cortisol levels. This region’s neuroplasticity is strongly linked to both the etiology of MDD and the effects of antidepressant treatments (Dahmen et al., 2018; Ren and Guo, 2021; Tartt et al., 2022).

**Figure 2:**
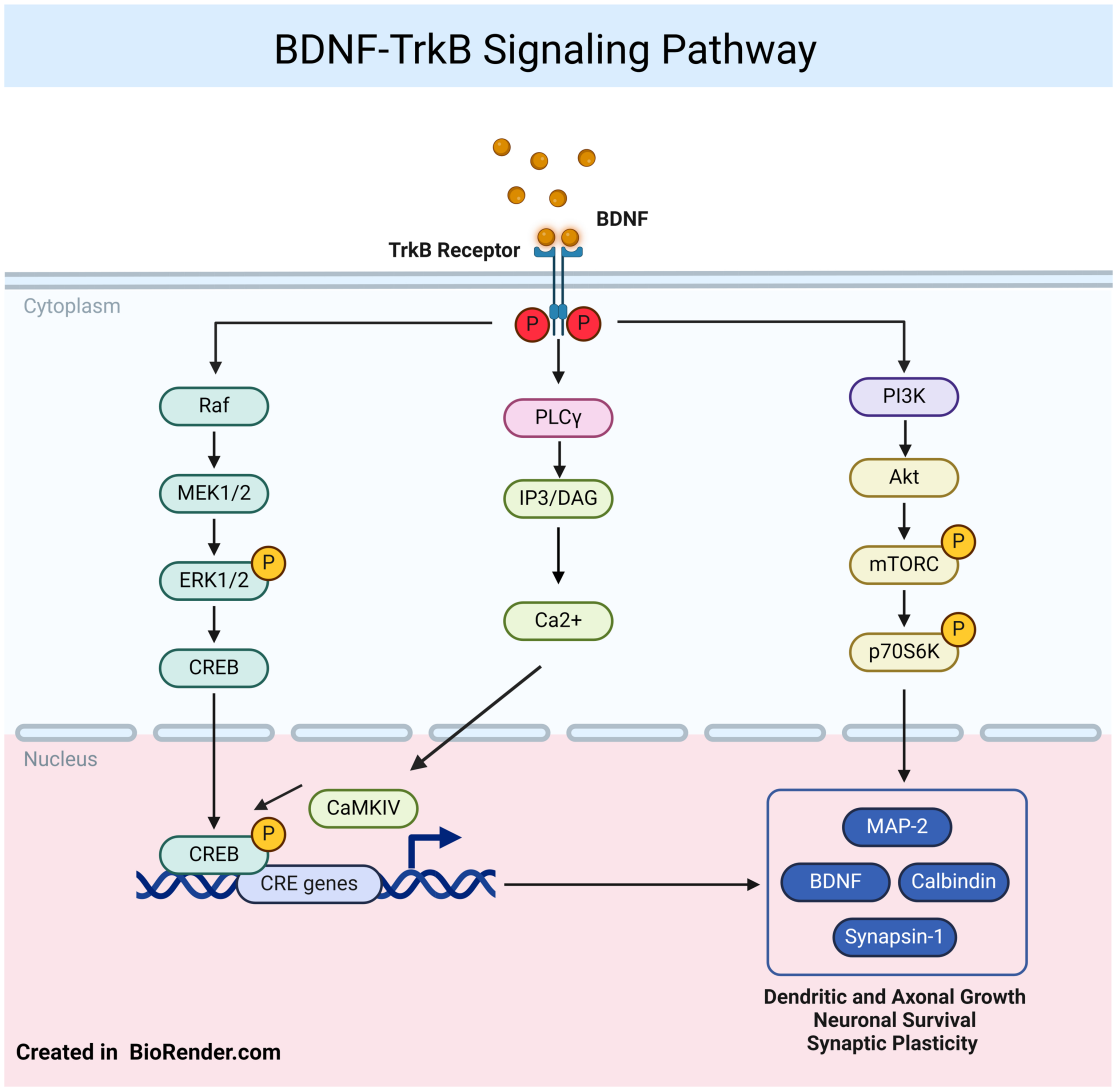
BDNF–TrkB signaling: the BDNF protein and its receptor TrkB participate in various signaling pathways, including phospholipase Cγ (PLCγ), phosphoinositide 3-kinase (PI3K), and mitogen-activated protein kinase/extracellular signal-regulated protein kinase (MAPK/ERK) pathways. Both ERK1/2 and cAMP response element binding protein (CREB) can translocate to the nucleus, where they phosphorylate CREB, promoting the transcription of genes involve in neuronal survival, dendritic growth, and synaptic plasticity. Figure was created using BioRender.com.

Another way to connect astrocyte function with neuroplasticity and depression is through the impact of certain astrocyte activation states on BDNF expression. Signaling through BDNF and its receptor TrkB (tropomyosin receptor kinase B) is essential for regulating neuronal survival, and structural and functional plasticity (Figure 2). BDNF released by astrocytes and neurons plays a significant role in neuronal function and astrocyte morphology, as it is critical for supporting synaptogenesis and neuronal communication. This is especially relevant in MDD, where impaired neuroplasticity is a hallmark, and enhancing BDNF signaling from astrocytes can improve synaptic health (Stahlberg et al., 2018). BDNF is implicated not only in the pathophysiology of depression but also in the therapeutic effects of antidepressants. For instance, in models of inflammation-related depression, the bacterial endotoxin LPS significantly decreases BDNF mRNA levels in the hippocampus following IL-1β or LPS injections (Zhang et al., 2016), with similar reductions in various cortical regions (Martínez-Turrillas et al., 2005). Studies have also found decreased expression of neurotrophins, such as BDNF, TrkB, nerve growth factor (NGF), and its receptor TrkA, at both protein and mRNA levels in the postmortem brains of suicide victims, emphasizing the role of these factors in the pathophysiology of depression and related behaviors (Erbay et al., 2021).

This highlights that astrocytes play an intricate role in supportive effects on neuroplasticity and neuronal function. Therefore, targeting the A1/A2 astrocytic balance and enhancing BDNF signaling within astrocytes provide a promising therapeutic strategy for treating major depressive disorder and other conditions where neuroplasticity is impaired. By promoting the neuroprotective A2 phenotype and increasing BDNF expression, it may be possible to counteract the detrimental effects of chronic stress and depression on the brain’s ability to recover and achieve homeostatic regulation.

## 4 Antidepressants and astrocytic function

Given the impact of astrocytic function on the pathophysiological mechanisms related to depression, it is expected that some pharmacological strategies used as conventional treatments for the disorder also affect astrocytes. Different antidepressants, such as selective serotonin reuptake inhibitors (SSRIs) and serotonin-norepinephrine reuptake inhibitors (SNRIs), act by restoring glial cell function and mitigating neuroinflammation. The ability of antidepressants to alleviate depressive behaviors and impact astrocytic phenotype and function has been demonstrated in various animal models. For example, in the Flinders Sensitive Line (FSL) rat model, ketamine reduced immobility and had a rapid and significant effect on astrocyte soma size and arborization (Ardalan et al., 2017). Similarly, it has been described that some SSRIs could improve altered behaviors by inhibiting the activation of neurotoxic A1 astrocytes (Fang et al., 2022a).

There is evidence showing that antidepressants reduce pro-inflammatory cytokine levels while restore or increase anti-inflammatory proteins and growth factors. For instance, fluoxetine has been linked to reduced IL-2 levels, and along with ketamine, it has been shown to reestablish the expression of glial cell line-derived neurotrophic factor (GDNF) and BDNF (Henkel et al., 2014; Kinoshita et al., 2018; Viana et al., 2020; Ma et al., 2022; Zeb et al., 2022). Additionally, dimethyl fumarate (DMF) has been proposed as a neuroprotective agent due to its ability to reverse increased Iba1 (ionized calcium-binding adapter molecule 1), TNF-*α*, and IL-1β expression, while counteracting the decrease in GFAP levels observed in CMS models (de Souza et al., 2022). Other antidepressants, such as paroxetine, sertraline, citalopram, fluvoxamine, and venlafaxine, have demonstrated inhibition of IL-1β and IL-6 expression (He et al., 2021). Notably, trazodone has been shown to reduce IL-6 levels, with a tendency to lower IFN-*γ* (interferon gamma) and increase IL-10 levels (Daniele et al., 2015). Moreover, the accumulation of α-synuclein is associated with astrogliosis, and some studies suggest that antidepressants reduce the number of α-syn-positive astrocytes, inhibiting α-syn propagation or inducing α-syn degradation by astroglia cells (Valera et al., 2014).

The effects of antidepressants on astrocyte function extend beyond regulating pro-inflammatory proteins. Neurodegeneration linked to neuroinflammation has been associated with lipid accumulation (Hansen and Wang, 2023). In this context, a study on primary rat cortical astrocytes showed that fluoxetine regulates lipid and amino acid metabolism and promotes synaptic plasticity. This suggests that fluoxetine’s antidepressant effects may involve regulating fatty acid and cholesterol metabolism through the activation of SREBP (Sterol Regulatory Element-Binding Protein) transcription factors (Bai et al., 2015). Furthermore, fluoxetine has been shown to promote autophagosome formation and remove damaged mitochondria in astrocytes in a CMS model, providing protection through a p53-dependent mechanism (Shu et al., 2019). Alongside amitriptyline and duloxetine, fluoxetine has also been studied for its effects on connexin 43 (Cx43) channels in astrocytes, which are involved in neurotransmitter regulation and are downregulated in depressive states. These antidepressants inhibited Cx43 hemichannel activity, showing differential effects on Cx43 intercellular junction channels, though they did not affect connexin expression levels (Koulakoff et al., 2008). Interestingly, another study demonstrated that fluoxetine inhibited A1 astrocyte activation via the astrocytic 5-HT2BR/*β*-arrestin2 pathway, improving depression and anxiety-like behaviors in CMS mouse models (Fang et al., 2022a). Additionally, this SSRI alleviated depression-like symptoms by increasing LC3 (Microtubule-associated protein 1A/1B-light chain 3) expression and autophagosome formation in the hippocampus, which promoted impaired mitochondrial clearance. These findings suggest that regulating autophagy may be another mode of action for fluoxetine, aside from its role in serotonin modulation (Shu et al., 2019).

Another antidepressant, mirtazapine, which is both noradrenergic and specifically serotonergic, has been shown to induce astrocyte proliferation and upregulate metallothionein, a protein involved in protecting against oxidative stress, via 5-HT1A receptors (5-HT1AR) on astrocytes (Kikuoka et al., 2020). Similarly, brexpiprazole, a partial 5-HT1A receptor agonist, was found to downregulate 5-HT1A and 5-HT7 receptors (5-HT7R) (Fukuyama et al., 2022). Despite these beneficial effects, antidepressants can also negatively affect astrocyte function. For example, sertraline and paroxetine have been shown to cause mitochondrial membrane damage, leading to reduced ATP production and increased reactive oxygen species (ROS) generation in astrocytes (Then et al., 2017).

Besides the therapeutic effect of common antidepressants, it has been reported that some immunomodulatory drugs can also reduce depressive symptoms. In a recent meta-analysis of randomized controlled trials, Bai et al. (2020) reported that anti-inflammatory agents (including Non-Steroidal Anti-Inflammatory Drugs – NSAIDs, polyunsaturated fatty acids, cytokine inhibitors, statins, corticosteroids, minocycline, pioglitazone, modafinil and *N*-acetylcysteine – NAC) could exert antidepressant effects and impact response and remission rates in MDD patients (Bai et al., 2020). Similarly, it has been found that patients exhibiting depressive symptoms associated with primary inflammatory disorders benefit from cytokine inhibitor treatment regardless of improvements in general physical health (Wittenberg et al., 2020).

In summary, these studies provide additional evidence of the strong connection between inflammatory responses and depression, highlighting that immune modulation is a promising pathway for further exploration. More specifically, it is emphasized that pharmacological agents preventing or reversing inflammation-induced reactive astrogliosis could effectively reduce depressive symptoms (Figure 1). However, the potential of these drugs to regulate inflammatory modulators through their impact on astrocyte reactivity remains underexplored (Dolotov et al., 2022). Besides, there is still a need for more detailed information on how different classes of antidepressants specifically influence astrocytic activity. This must include not only traditional drugs, like SSRIs and SNRIs, but also novel therapeutic approaches. Now, given the great versatility of astrocytes under different conditions, both physiological and pathological, and considering the increased research on these state transitions in the context of various disorders, it will be crucial to adopt a unified nomenclature to refer to these different astrocytic states. This nomenclature, rather than being a simplistic classification, should be more specific and based on transcriptomic, proteomic, metabolic, and other profiles characteristic of astrocytes under different conditions (Escartin et al., 2021). This approach toward a unified nomenclature in future research will be essential to guide efforts aligned with a joint pursuit of increasing understanding of the disorder’s pathophysiology, as well as refining the development of therapeutic strategies.

## 5 Conclusion

This mini-review highlights the essential role of astrocytes in the development and persistence of major depressive disorder, especially through their involvement in neuroinflammation and the regulation of neuroplasticity. Although there are valuable insights into the function of astrocytes in depression, significant gaps persist, particularly concerning the specific mechanisms by which astrocyte dysfunction contributes to depressive symptoms. This understanding would be essential for developing more assertive treatment strategies. One potential therapeutic approach could involve targeting different astrocyte states, since modulating the balance between phenotypes related to neuroprotection and those more typical of neuroinflammation responses may offer promising new strategies for treatment. In particular, to promote the astrocyte-mediated neuroplasticity process, could improve brain resilience, and reduce the neuronal damage often observed in patients with depression.

Furthermore, the use of classic psychedelics, which have shown potential in treating mood disorders, remains an area that has not been fully explored in terms of their effects on astrocyte reactivity (VanderZwaag et al., 2023). Understanding how these substances interact with astrocytes may not only provide new insights into their therapeutic mechanisms but could also expand the range of treatment options for mood disorders. Future research should focus on these promising areas to develop new, astrocyte-targeted therapies, that could better address the underlying causes of depression and improve patient outcomes.

## References

[ref1] Ardalan M. Rafati A. H. Nyengaard J. R. Wegener G. (2017). Rapid antidepressant effect of ketamine correlates with astroglial plasticity in the hippocampus. Br. J. Pharmacol. 174, 483–492. doi: 10.1111/bph.13714, PMID: 28087979 PMC5323512

[ref2] Aten S. Du Y. Taylor O. Dye C. Collins K. Thomas M.. (2023). Chronic stress impairs the structure and function of astrocyte networks in an animal model of depression. Neurochem. Res. 48, 1191–1210. doi: 10.1007/s11064-022-03663-4, PMID: 35796915 PMC9823156

[ref3] Bai S. Guo W. Feng Y. Deng H. Li G. Nie H.. (2020). Efficacy and safety of anti-inflammatory agents for the treatment of major depressive disorder: a systematic review and meta-analysis of randomised controlled trials. J. Neurol. Neurosurg. Psychiatry 91:912. doi: 10.1136/jnnp-2019-320912 31658959

[ref4] Bai S. Zhou C. Cheng P. Fu Y. Fang L. Huang W.. (2015). 1H NMR-based metabolic profiling reveals the effects of fluoxetine on lipid and amino acid metabolism in astrocytes. Int. J. Mol. Sci. 16, 8490–8504. doi: 10.3390/ijms16048490, PMID: 25884334 PMC4425092

[ref5] Banasr M. Duman R. S. (2008). Glial loss in the prefrontal cortex is sufficient to induce depressive-like behaviors. Biol. Psychiatry 64:8. doi: 10.1016/j.biopsych.2008.06.008 PMC2709733 18639237

[ref6] Bélanger M. Allaman I. Magistretti P. J. (2011). Brain energy metabolism: focus on astrocyte-neuron metabolic cooperation. Cell Metab. 14, 724–738. doi: 10.1016/j.cmet.2011.08.016, PMID: 22152301

[ref7] Cao B. Passos I. C. Mwangi B. Amaral-Silva H. Tannous J. Wu M. J.. (2017). Hippocampal subfield volumes in mood disorders. Mol. Psychiatry 22, 1352–1358. doi: 10.1038/mp.2016.262, PMID: 28115740 PMC5524625

[ref8] Chang J. Qian Z. Wang B. Cao J. Zhang S. Jiang F.. (2023). Transplantation of A2 type astrocytes promotes neural repair and remyelination after spinal cord injury. Cell Commun. Signal 21:37. doi: 10.1186/s12964-022-01036-6 36797790 PMC9936716

[ref9] Clarke L. E. Liddelow S. A. Chakraborty C. Münch A. E. Heiman M. Barres B. A. (2018). Normal aging induces A1-like astrocyte reactivity. Proc. Natl. Acad. Sci. USA 115, E1896–E1905. doi: 10.1073/pnas.1800165115, PMID: 29437957 PMC5828643

[ref10] Cosgrove L. Brhlikova P. Lyus R. Herrawi F. D’Ambrozio G. Abi-Jaoude E.. (2024). Global burden disease estimates for major depressive disorders (MDD): a review of diagnostic instruments used in studies of prevalence. Community Ment. Health J. doi: 10.1007/s10597-024-01338-8, PMID: 39133359

[ref11] Cui L. Li S. Wang S. Wu X. Liu Y. Yu W.. (2024). Major depressive disorder: hypothesis, mechanism, prevention and treatment. US: Springer. 10.1038/s41392-024-01738-y PMC10853571 38331979

[ref12] Dahmen B. Puetz V. B. Scharke W. von Polier G. G. Herpertz-Dahlmann B. Konrad K. (2018). Effects of early-life adversity on hippocampal structures and associated HPA Axis functions. Dev. Neurosci. 40, 13–22. doi: 10.1159/000484238, PMID: 29237154

[ref13] Daniele S. Zappelli E. Martini C. (2015). Trazodone regulates neurotrophic/growth factors, mitogen-activated protein kinases and lactate release in human primary astrocytes. J. Neuroinflammation 12:225. doi: 10.1186/s12974-015-0446-x 26627476 PMC4666178

[ref14] de Souza A. G. Lopes I. S. Filho A. J. M. C. Cavalcante T. M. B. Oliveira J. V. S. de Carvalho M. A. J.. (2022). Neuroprotective effects of dimethyl fumarate against depression-like behaviors via astrocytes and microglia modulation in mice: possible involvement of the HCAR2/Nrf2 signaling pathway. Naunyn Schmiedeberg’s Arch. Pharmacol. 395, 1029–1045. doi: 10.1007/s00210-022-02247-x, PMID: 35665831

[ref15] Dolotov O. V. Inozemtseva L. S. Myasoedov N. F. Grivennikov I. A. (2022). Stress-induced depression and Alzheimer’s disease: focus on astrocytes. Int. J. Mol. Sci. 23:4999. doi: 10.3390/ijms23094999, PMID: 35563389 PMC9104432

[ref16] Erbay L. G. Karlıdağ R. Oruç M. Çiğremiş Y. Celbiş O. (2021). Association of BDNF/TrkB and NGF/TrkA levels in postmortem brain with major depression and suicide. Psychiatr. Danub. 33, 491–498. doi: 10.24869/psyd.2021.491, PMID: 34928896

[ref17] Escartin C. Galea E. Lakatos A. O’Callaghan J. P. Petzold G. C. Serrano-Pozo A.. (2021). Reactive astrocyte nomenclature, definitions, and future directions. Nat. Neurosci. 24, 312–325. doi: 10.1038/s41593-020-00783-4, PMID: 33589835 PMC8007081

[ref18] Fang Y. Ding X. Zhang Y. Cai L. Ge Y. Ma K.. (2022a). Fluoxetine inhibited the activation of A1 reactive astrocyte in a mouse model of major depressive disorder through astrocytic 5-HT. J. Neuroinflammation 19:23. doi: 10.1186/s12974-022-02389-y 35093099 PMC8800238

[ref19] Fang Y. Guo H. Wang Q. Liu C. Ge S. Yan B. (2022b). The role and mechanism of NLRP3 inflammasome-mediated astrocyte activation in dehydrocorydaline against CUMS-induced depression. Front. Pharmacol. 13:1008249. doi: 10.3389/fphar.2022.1008249 36506556 PMC9726715

[ref20] Fukuyama K. Motomura E. Okada M. (2022). Brexpiprazole reduces 5-HT7 receptor function on astroglial transmission systems. Int. J. Mol. Sci. 23:6571. doi: 10.3390/ijms23126571, PMID: 35743014 PMC9223571

[ref21] Giovannoni F. Quintana F. J. (2020). The role of astrocytes in CNS inflammation. Trends Immunol. 41:7. doi: 10.1016/j.it.2020.07.007 32800705 PMC8284746

[ref22] Gradisnik L. Velnar T. (2023). Astrocytes in the central nervous system and their functions in health and disease: a review. World J. Clin. Cases 11:3385. doi: 10.12998/wjcc.v11.i15.3385, PMID: 37383914 PMC10294192

[ref23] Hansen S. B. Wang H. (2023). The shared role of cholesterol in neuronal and peripheral inflammation. Pharmacol. Ther. 249:108486. doi: 10.1016/j.pharmthera.2023.108486, PMID: 37390970

[ref24] Hao T. Du X. Yang S. Zhang Y. Liang F. (2020). Astrocytes-induced neuronal inhibition contributes to depressive-like behaviors during chronic stress. Life Sci. 258:8099. doi: 10.1016/j.lfs.2020.118099 32682917

[ref25] He J. H. Liu R. P. Peng Y. M. Guo Q. Zhu L. B. Lian Y. Z.. (2021). Differential and paradoxical roles of new-generation antidepressants in primary astrocytic inflammation. J. Neuroinflammation 18:47. doi: 10.1186/s12974-021-02097-z 33602262 PMC7890881

[ref26] Henkel A. W. Alali H. Devassy A. Alawadi M. M. Redzic Z. B. (2014). Antagonistic interactions between dexamethasone and fluoxetine modulate morphodynamics and expression of cytokines in astrocytes. Neuroscience 280, 318–327. doi: 10.1016/j.neuroscience.2014.09.012, PMID: 25242644

[ref27] Hinkle J. T. Dawson V. L. Dawson T. M. (2019). The A1 astrocyte paradigm: new avenues for pharmacological intervention in neurodegeneration. Mov. Disord. 34, 959–969. doi: 10.1002/mds.27718, PMID: 31136698 PMC6642014

[ref28] Howren M. B. Lamkin D. M. Suls J. (2009). Associations of depression with c-reactive protein, IL-1, and IL-6: a meta-analysis. Psychosom. Med. 71, 171–186. doi: 10.1097/PSY.0b013e3181907c1b, PMID: 19188531

[ref29] James S. L. Abate D. Abate K. H. Abay S. M. Abbafati C. Abbasi N.. (2018). Global, regional, and national incidence, prevalence, and years lived with disability for 354 diseases and injuries for 195 countries and territories, 1990-2017: a systematic analysis for the global burden of disease study 2017. Lancet 392, 1789–1858. doi: 10.1016/S0140-6736(18)32279-7, PMID: 30496104 PMC6227754

[ref30] Kikuoka R. Miyazaki I. Kubota N. Maeda M. Kagawa D. Moriyama M.. (2020). Mirtazapine exerts astrocyte-mediated dopaminergic neuroprotection. Sci. Rep. 10:20698. doi: 10.1038/s41598-020-77652-4 33244123 PMC7693322

[ref31] Kim J. H. Suh S. I. Lee H. J. Lee J. H. Lee M. S. (2019). Cortical and subcortical gray matter alterations in first-episode drug-naïve adolescents with major depressive disorder. Neuroreport 30, 1172–1178. doi: 10.1097/WNR.0000000000001336, PMID: 31568197 PMC6855326

[ref32] Kim Y. K. Won E. (2017). The influence of stress on neuroinflammation and alterations in brain structure and function in major depressive disorder. Behav. Brain Res. 329, 6–11. doi: 10.1016/j.bbr.2017.04.020, PMID: 28442354

[ref33] Kinoshita M. Hirayama Y. Fujishita K. Shibata K. Shinozaki Y. Shigetomi E.. (2018). Anti-depressant fluoxetine reveals its therapeutic effect via astrocytes. EBioMedicine 32, 72–83. doi: 10.1016/j.ebiom.2018.05.036, PMID: 29887330 PMC6020856

[ref34] Kouba B. R. de Araujo Borba L. de Souza P. Gil-Mohapel J. Rodrigues A. L. S. (2024). Role of inflammatory mechanisms in major depressive disorder: from etiology to potential pharmacological targets. Cells 13:423. doi: 10.3390/cells13050423, PMID: 38474387 PMC10931285

[ref35] Koulakoff A. Ezan P. Giaume C. (2008). Neurons control the expression of connexin 30 and connexin 43 in mouse cortical astrocytes. Glia 56:698. doi: 10.1002/glia.20698 18512249

[ref36] Kuhn M. Höger N. Feige B. Blechert J. Normann C. Nissen C. (2014). Fear extinction as a model for synaptic plasticity in major depressive disorder. PLoS One 9:e115280. doi: 10.1371/journal.pone.0115280, PMID: 25545818 PMC4278908

[ref37] Leng L. Zhuang K. Liu Z. Huang C. Gao Y. Chen G.. (2018). Menin deficiency leads to depressive-like behaviors in mice by modulating astrocyte-mediated Neuroinflammation. Neuron 100:31. doi: 10.1016/j.neuron.2018.08.031 30220511

[ref38] Li S. Fang Y. Zhang Y. Song M. Zhang X. Ding X.. (2022). Microglial NLRP3 inflammasome activates neurotoxic astrocytes in depression-like mice. Cell Rep. 41:1532. doi: 10.1016/j.celrep.2022.111532 36288697

[ref39] Li Z. Ruan M. Chen J. Fang Y. (2021). Major depressive disorder: advances in neuroscience research and translational applications. Neurosci. Bull. 37, 863–880. doi: 10.1007/s12264-021-00638-3, PMID: 33582959 PMC8192601

[ref40] Li S. Sun Y. Song M. Song Y. Fang Y. Zhang Q.. (2021). NLRP3/caspase-1/GSDMD-mediated pyroptosis exerts a crucial role in astrocyte pathological injury in mouse model of depression. JCI Insight 6:852. doi: 10.1172/jci.insight.146852 PMC8675200 34877938

[ref41] Liu Y. Chen L. Lin L. Xu C. Xiong Y. Qiu H.. (2024). Unveiling the hidden pathways: exploring astrocytes as a key target for depression therapy. J. Psychiatr. Res. 174, 101–113. doi: 10.1016/j.jpsychires.2024.04.003, PMID: 38626560

[ref42] Liu L. R. Liu J. C. Bao J. S. Bai Q. Q. Wang G. Q. (2020). Interaction of microglia and astrocytes in the neurovascular unit. Front. Immunol. 11:1024. doi: 10.3389/fimmu.2020.01024 32733433 PMC7362712

[ref43] Liu R. T. Rowan-Nash A. D. Sheehan A. E. Walsh R. F. L. Sanzari C. M. Korry B. J.. (2020). Reductions in anti-inflammatory gut bacteria are associated with depression in a sample of young adults. Brain Behav. Immun. 88:26. doi: 10.1016/j.bbi.2020.03.026 PMC7415740 32229219

[ref44] Lu C. L. Ren J. Mo J. W. Fan J. Guo F. Chen L. Y.. (2022). Glucocorticoid receptor–dependent astrocytes mediate stress vulnerability. Biol. Psychiatry 92:22. doi: 10.1016/j.biopsych.2021.11.022 35151464

[ref45] Ma X. Yang S. Zhang Z. Liu L. Shi W. Li S.. (2022). Rapid and sustained restoration of astrocytic functions by ketamine in depression model mice. Biochem. Biophys. Res. Commun. 616, 89–94. doi: 10.1016/j.bbrc.2022.03.068, PMID: 35653826

[ref46] Maeng S. H. Hong H. (2019). Inflammation as the potential basis in depression. Int. Neurourol. J. 23:113. doi: 10.5213/inj.1938226.113 PMC6905209 31795605

[ref47] Martínez-Turrillas R. Del Río J. Frechilla D. (2005). Sequential changes in BDNF mRNA expression and synaptic levels of AMPA receptor subunits in rat hippocampus after chronic antidepressant treatment. Neuropharmacology 49, 1178–1188. doi: 10.1016/j.neuropharm.2005.07.006, PMID: 16143352

[ref48] Milaneschi Y. Kappelmann N. Ye Z. Lamers F. Moser S. Jones P. B.. (2021). Association of inflammation with depression and anxiety: evidence for symptom-specificity and potential causality from UK biobank and NESDA cohorts. Mol. Psychiatry 26, 7393–7402. doi: 10.1038/s41380-021-01188-w, PMID: 34135474 PMC8873022

[ref49] Munshi S. Loh M. K. Ferrara N. DeJoseph M. R. Ritger A. Padival M.. (2020). Repeated stress induces a pro-inflammatory state, increases amygdala neuronal and microglial activation, and causes anxiety in adult male rats. Brain Behav. Immun. 84:23. doi: 10.1016/j.bbi.2019.11.023 31785394 PMC7010555

[ref50] Novakovic M. M. Korshunov K. S. Grant R. A. Martin M. E. Valencia H. A. Budinger G. R. S.. (2023). Astrocyte reactivity and inflammation-induced depression-like behaviors are regulated by Orai1 calcium channels. Nat. Commun. 14:5500. doi: 10.1038/s41467-023-40968-6 37679321 PMC10485021

[ref51] Peng L. Verkhratsky A. Gu L. Li B. (2015). Targeting astrocytes in major depression. Expert. Rev. Neurother. 15:5094. doi: 10.1586/14737175.2015.1095094 26471936

[ref52] Planas-Fontánez T. M. Dreyfus C. F. Saitta K. S. (2020). Reactive astrocytes as therapeutic targets for brain degenerative diseases: roles played by metabotropic glutamate receptors. Neurochem. Res. 45, 541–550. doi: 10.1007/s11064-020-02968-6, PMID: 31983009 PMC7058558

[ref53] Ren F. Guo R. (2021). Synaptic microenvironment in depressive disorder: insights from synaptic plasticity. Neuropsychiatr. Dis. Treat. 17, 157–165. doi: 10.2147/NDT.S268012 33519203 PMC7838013

[ref54] Schmaal L. Hibar D. P. Sämann P. G. Hall G. B. Baune B. T. Jahanshad N.. (2017). Cortical abnormalities in adults and adolescents with major depression based on brain scans from 20 cohorts worldwide in the ENIGMA major depressive disorder working group. Mol. Psychiatry 22, 900–909. doi: 10.1038/mp.2016.60, PMID: 27137745 PMC5444023

[ref55] Shu X. Sun Y. Sun X. Zhou Y. Bian Y. Shu Z.. (2019). The effect of fluoxetine on astrocyte autophagy flux and injured mitochondria clearance in a mouse model of depression. Cell Death Dis. 10:577. doi: 10.1038/s41419-019-1813-9 31371719 PMC6675792

[ref56] Sofroniew M. V. (2020). Astrocyte reactivity: subtypes, states, and functions in CNS innate immunity. Trends Immunol. 41, 758–770. doi: 10.1016/j.it.2020.07.004, PMID: 32819810 PMC7484257

[ref57] Stahlberg M. A. Kügler S. Dean C. (2018). Visualizing BDNF cell-to-cell transfer reveals astrocytes are the primary recipient of neuronal BDNF. bioRxiv:255935. doi: 10.1101/255935

[ref58] Tartt A. N. Mariani M. B. Hen R. Mann J. J. Boldrini M. (2022). Dysregulation of adult hippocampal neuroplasticity in major depression: pathogenesis and therapeutic implications. Mol. Psychiatry 27, 2689–2699. doi: 10.1038/s41380-022-01520-y, PMID: 35354926 PMC9167750

[ref59] Then C. K. Liu K. H. Liao M. H. Chung K. H. Wang J. Y. Shen S. C. (2017). Antidepressants, sertraline and paroxetine, increase calcium influx and induce mitochondrial damage-mediated apoptosis of astrocytes. Oncotarget 8, 115490–115502. doi: 10.18632/oncotarget.23302, PMID: 29383176 PMC5777788

[ref60] Turati J. Ramírez D. Carniglia L. Saba J. Caruso C. Quarleri J.. (2020). Antioxidant and neuroprotective effects of mGlu3 receptor activation on astrocytes aged in vitro. Neurochem. Int. 140:4837. doi: 10.1016/j.neuint.2020.104837 32858088

[ref61] Valera E. Ubhi K. Mante M. Rockenstein E. Masliah E. (2014). Antidepressants reduce neuroinflammatory responses and astroglial alpha-synuclein accumulation in a transgenic mouse model of multiple system atrophy. Glia 62, 317–337. doi: 10.1002/glia.22610, PMID: 24310907 PMC4183229

[ref62] VanderZwaag J. Halvorson T. Dolhan K. Šimončičová E. Ben-Azu B. Tremblay M. È. (2023). The missing piece? A case for Microglia’s prominent role in the therapeutic action of anesthetics, ketamine, and psychedelics. Neurochem. Res. 48, 1129–1166. doi: 10.1007/s11064-022-03772-0, PMID: 36327017

[ref63] Viana G. S. B. Vale E. M. D. Araujo A. R. A. Coelho N. C. Andrade S. M. Costa R. O. D.. (2020). Rapid and long-lasting antidepressant-like effects of ketamine and their relationship with the expression of brain enzymes, BDNF, and astrocytes. Braz. J. Med. Biol. Res. 54:e10107. doi: 10.1590/1414-431X202010107, PMID: 33331415 PMC7747878

[ref64] Wang Q. Jie W. Liu J. H. Yang J. M. Gao T. M. (2017). An astroglial basis of major depressive disorder? An overview. Glia 65:3143. doi: 10.1002/glia.23143 28317185

[ref65] Wang Y. Ni J. Zhai L. Gao C. Xie L. Zhao L.. (2019). Inhibition of activated astrocyte ameliorates lipopolysaccharide-induced depressive-like behaviors. J. Affect. Disord. 242:15. doi: 10.1016/j.jad.2018.08.015 30172225

[ref66] Wittenberg G. M. Stylianou A. Zhang Y. Sun Y. Gupta A. Jagannatha P. S.. (2020). Effects of immunomodulatory drugs on depressive symptoms: a mega-analysis of randomized, placebo-controlled clinical trials in inflammatory disorders. Mol. Psychiatry 25, 1275–1285. doi: 10.1038/s41380-019-0471-8, PMID: 31427751 PMC7244402

[ref67] World Health Organization. (2021). Depression. Available at: https://www.who.int/news-room/fact-sheets/detail/depression (accessed November 5, 2021).

[ref68] Yao J. Chen C. Guo Y. Yang Y. Liu X. Chu S.. (2023). A review of research on the association between neuron–astrocyte signaling processes and depressive symptoms. Int. J. Mol. Sci. 24:6985. doi: 10.3390/ijms24086985, PMID: 37108148 PMC10139177

[ref69] Zeb S. Ye H. Liu Y. Du H. P. Guo Y. Zhu Y. M.. (2022). Necroptotic kinases are involved in the reduction of depression-induced astrocytes and fluoxetine’s inhibitory effects on necroptotic kinases. Front. Pharmacol. 13:1060954. doi: 10.3389/fphar.2022.1060954, PMID: 36686688 PMC9847570

[ref70] Zhang D. Hua Z. Li Z. (2024). The role of glutamate and glutamine metabolism and related transporters in nerve cells. CNS Neurosci. Ther. 30:e14617. doi: 10.1111/cns.14617, PMID: 38358002 PMC10867874

[ref71] Zhang J. C. Yao W. Hashimoto K. (2016). Brain-derived neurotrophic factor (BDNF)-TrkB signaling in inflammation-related depression and potential therapeutic targets. Curr. Neuropharmacol. 14, 721–731. doi: 10.2174/1570159X14666160119094646, PMID: 26786147 PMC5050398

